# Nucleus Accumbens Resting State Functional Connectivity is Linked to Family Income, Reward Salience, and Substance Use

**DOI:** 10.31586/jcn.2025.1244

**Published:** 2025-02-25

**Authors:** Shervin Assari, Alexandra Donovan

**Affiliations:** 1 Department of Internal Medicine, Charles R. Drew University of Medicine and Science, Los Angeles, CA, United States; 2 Department of Family Medicine, Charles R. Drew University of Medicine and Science, Los Angeles, CA, United States; 3 Department of Urban Public Health, Charles R. Drew University of Medicine and Science, Los Angeles, CA, United States; 4 Marginalization-Related Diminished Returns (MDRs) Center, Los Angeles, CA, United States

**Keywords:** Nucleus Accumbens (NAcc), Resting-State Functional Connectivity (rsFC), Socioeconomic Status (SES), Reward Salience, Substance Use, Adolescence

## Abstract

**Background:**

As a central component of the brain’s reward system, nucleus accumbens (NAcc) plays a crucial role in reward salience and substance use behaviors. Changes in the NAcc are also relevant to higher rates of substance use of youth and adults from low-income backgrounds. Although resting-state functional connectivity (rsFC) of the NAcc provides valuable insights into the neural mechanisms underlying reward processing and the propensity for self-reported reward salience and substance use, research exploring the association between NAcc rsFC and brain networks beyond the default mode network (DMN) and prefrontal cortex (PFC) is limited.

**Objective:**

To investigate the role of the resting-state functional connectivity of the NAcc with the cingulo-opercular network, sensorimotor mouth network, and sensorimotor hand network in the association between socioeconomic status, self-reported reward salience, and future substance use.

**Methods:**

Data were obtained from the Adolescent Brain Cognitive Development (ABCD) study. NAcc rsFC with the cingulo-opercular network, sensorimotor mouth network, and sensorimotor hand network was assessed at baseline. Socioeconomic status was measured using family income. Self-reported reward salience was assessed using validated psychometric scales. Substance use outcomes were tracked longitudinally over the study period. Structural Equation Modeling was employed to examine the covariances between family income, NAcc rsFC, reward salience, and subsequent substance use.

**Results:**

Higher baseline family income was positively associated with baseline NAcc rsFC (B = 0.092, p < 0.001) and negatively associated with baseline reward salience (B = −0.040, p = 0.036) and future substance use (B = −0.081, p < 0.001). Baseline NAcc rsFC was strongly and positively associated with reward salience (B = 0.734, p < 0.001) and future substance use up to age 13 (B = 0.124, p < 0.001). Additionally, baseline reward salience was positively associated with future substance use (Covariance = 0.176, p < 0.001).

**Conclusion:**

The findings suggest that NAcc rsFC with brain networks beyond the DMN or PFC may contribute to the links between low parental socioeconomic status, reward salience, and substance use risk. Expanding the understanding of NAcc rsFC provides new insights into the neural mechanisms underlying these associations. These results have important implications for developing targeted interventions aimed at preventing substance use, particularly among low-income youth with heightened reward salience. Further research is needed to explore causal pathways and moderating factors influencing these relationships.

## 1. Introduction

Youth substance use is a prevalent concern [1], with significant long-term consequences that can extend into adulthood, affecting physical health, mental well-being, and socioeconomic outcomes [2–4]. Among adolescents, those from low-income backgrounds face an even higher risk of substance use[5]. This elevated risk can be attributed to the interplay between environmental stressors and neurobiological vulnerabilities. Neurodevelopmental processes [6,7], including heightened activity and sensitivity of the brain’s reward system, make adolescence a critical period for developing health behaviors [8–11]. This leaves adolescents more responsive to rewarding stimuli, such as substances, than adults.

The nucleus accumbens (NAcc) plays a pivotal role in reward processing in the brain, serving as a central hub in the mesocorticolimbic dopamine system [12–14]. It integrates signals from cortical and limbic regions, mediating responses to rewarding stimuli, including food, social interactions, and addictive substances [15,16]. Substances such as nicotine and marijuana exploit the NAcc by artificially enhancing dopamine release, creating a powerful cycle of reward-seeking behavior that can lead to dependence [27]. The NAcc’s dopaminergic inputs make it a critical player in reinforcing behaviors, which contribute to the development and persistence of substance use [15,17–21]. Dysregulation of NAcc activity, whether through heightened activity or reduced inhibitory control, is associated with increased vulnerability to substance use [22–27]. Additionally, NAcc structure and function predicts future substance use [26] and obesity [28] in adolescents. Thus, understanding how other factors associated with increased substance use affect NAcc during reward processing would provide crucial insights into the neurobiological underpinnings of substance use and addiction.

The brain operates as a dynamic network of spatially distributed yet functionally interconnected regions that continuously share information [29–31]. Advances in resting-state functional connectivity (rsFC) using functional magnetic resonance imaging (fMRI) have enabled researchers to explore the temporal synchronization of neuronal activity between anatomically distinct brain regions [32]. For example, a recent study of Mexican-origin adolescents explored the relationship between the age of substance use onset and rsFC between the NAcc and the prefrontal cortex (PFC) and other regions involved in cognitive control [34]. Earlier initiation of substance use was associated with stronger connectivity between the bilateral NAcc and several regions, including the right dorsolateral PFC, right dorsomedial PFC, right pre-supplementary motor area, right inferior parietal lobule, and left medial temporal gyrus. These brain regions are part of the right frontoparietal network, which plays a critical role in cognitive control. The observed coupling between reward and cognitive control networks may represent a mechanism by which earlier substance use onset influences brain function over time, potentially contributing to the development of substance use disorders later in life. This is surprising considering one would expect lower connectivity between PFC-NAcc to lead to earlier substance initiation [34].

This approach has significant implications for understanding the neural mechanisms that contribute to substance use disorders and developing interventions to mitigate these risks during adolescence—a critical developmental window when the reward system is particularly active and susceptible to environmental influences.

This study was conducted to investigate the role of the resting-state functional connectivity of the NAcc with the cingulo-opercular network (CON), sensorimotor mouth network (SMM), and sensorimotor hand (SMH) network in the association between socioeconomic status (SES), self-reported reward salience, and future substance use.

## 2. Methods

### 2.1. Study Design and Participants

This study utilized data from the Adolescent Brain Cognitive Development (ABCD) Study, a longitudinal, multisite cohort study designed to explore factors influencing brain development and health in youth across the United States. We analyzed baseline data collected from a diverse sample of children aged 9–10 years, focusing on demographic, social, and neuroimaging variables. The study population included children from a range of racial/ethnic backgrounds and socioeconomic contexts.

The study had a balanced number of male and female participants, and no exclusion criteria were applied; participants were included regardless of socioeconomic status (SES), race/ethnicity, or handedness. However, for the tobacco use variable, about 100 children who had already experimented with tobacco at baseline were excluded, as our focus was on subsequent tobacco initiation.

### 2.2. Measures

#### 2.2.1. Resting-State Functional Connectivity (rsFC)

The MRI procedures used in the ABCD Study are thoroughly explained in detail in other publications [36–41]. The ABCD Imaging Acquisition Workgroup (https://abcdstudy.org/scientists-workgroups.html) developed, refined, and standardized the imaging measures and protocols across all 21 ABCD sites. This referenced work outlines the foundation and methodology of the ABCD imaging protocols and provides an initial assessment of their quality, demonstrating their suitability for children aged 9 to 10 years [42]. The ABCD team calculated rsFC values using standard preprocessing pipelines, including motion correction, spatial normalization, and filtering, to derive connectivity metrics. NAcc rsFC was quantified as the Fisher’s z-transformed correlation between mean time series of the two networks.

#### 2.2.2. Substance Use (Tobacco and Marijuana Use)

Tobacco and marijuana use were assessed every six months. At baseline (Y0), participants provided information about their lifetime substance use through an online Timeline Follow-Back (TLFB) interview [43,44]. Subsequent assessments reported on use within the past six months or since the last assessment of use. The study examined a variety of substances, with additional data collected during semi-annual telephone follow-ups to capture yearly substance use patterns. For this study, substance use was categorized into experimental use (e.g., limited tobacco or marijuana use) and initiation (defined as more than one instance of use). To track the onset of tobacco and marijuana use occurring at least six months after the study began, two variables were created to facilitate analysis. In this study, “tobacco” includes any tobacco product, such as traditional cigarettes and e-cigarettes. We also derived a latent factor representing the initiation of both tobacco and marijuana use.

#### 2.2.3. Reward Salience (RR and FS).

In this study, fun-seeking and reward responsiveness were assessed using the Behavioral Approach System (BAS), derived from Carver’s model within the framework of the reinforcement sensitivity theory (RST) [45]. This construct captures reward-seeking tendencies, traits that are strongly associated with impulsivity and risk-taking behaviors. Fun-seeking and reward responsiveness have been linked to various high-risk activities, including tobacco use, alcohol consumption, emotional eating, obesity, aggression, and sexual risk behaviors [46]. According to Gray’s reinforcement sensitivity theory [47,48], individuals with high reward salience are particularly attuned to conditioned cues that signal an increased likelihood of reward. For this analysis, fun-seeking was measured as a continuous variable, operationalized through BAS-based assessments of reward salience.

#### 2.2.4. Family Income

Family income was assessed using a 1–10 scale, where a higher score indicated a higher income level. Participants reported the total family income over the past 12 months, with the following options: 1 = less than $5,000; 2 = $5,000; 3 = $12,000; 4 = $16,000; 5 = $25,000; 6 = $35,000; 7 = $50,000; 8 = $75,000; 9 = $100,000; and 10 = $200,000. Appendix B provides a detailed summary of this income variable.

### 2.3. Statistical Analysis

Structural equation modeling (SEM) [52] was employed to examine the relationships between reward salience, rsFC, family income, and substance use. Reward salience, rsFC, and substance use were treated as latent factors. In our study, family income was an observed variable. Model coefficients (B), standard errors (SE), and p-values were reported for all paths. Model fit was assessed using the Comparative Fit Index (CFI), Tucker-Lewis Index (TLI), and Root Mean Square Error of Approximation (RMSEA) [53]. A CFI and TLI above 0.90 and an RMSEA below 0.08 indicated acceptable model fit. Missing data were handled using full information maximum likelihood estimation. All analyses were conducted using Stata 18.0.

### 2.4. Ethical Considerations

The ABCD Study protocol was approved by the institutional review board of the UCSD. Informed consent and assent were obtained from parents and children, respectively. Data were analyzed anonymously.

## 3. Results

Table 1 provides the descriptive statistics for the study variables. The mean family income was 7.213 (SE = 0.028), indicating a relatively stable socioeconomic background among the participants (between $50,000 and $75,000 annually). Reward responsiveness had a mean of 10.963 (SE = 0.034), and fun seeking exhibited a mean of 5.667 (SE = 0.031), reflecting moderate levels of reward salience in the sample. Regarding NAcc rsFC with specific brain networks, the mean rsFC with the right SMH network was 0.209 (SE = 0.002). The mean rsFC with the left CON network was 0.098 (SE = 0.002), while the mean rsFC with the left sensorimotor mouth (SMM) network was 0.195 (SE = 0.003). These values indicate modest connectivity levels between the NAcc and the specified brain networks.

Table 2 summarizes the bivariate associations among family income, NAcc rsFC components, reward salience components (reward responsiveness and fun seeking), and future substance use (tobacco and marijuana use). Family income was negatively associated with reward responsiveness (r = −0.102, p < 0.001) and fun seeking (r = −0.091, p < 0.001), indicating that lower income is related to higher reward salience. Family income showed a positive association with rsFC in the right SMH (r = 0.110, p < 0.001) and left CON (r = 0.053, p < 0.001). Conversely, family income had a small but significant negative relationship with future tobacco use (r = −0.046, p < 0.001) and future marijuana use (r = −0.066, p < 0.001). Reward responsiveness was positively associated with fun seeking (r = 0.467, p < 0.001) but showed a weak negative association with rsFC in the right SMH region (r = −0.020, p = 0.075). Fun seeking also demonstrated small negative associations with rsFC in the right SMH (r = −0.049, p < 0.001) and left CON (r = −0.009, p = 0.421), although the latter was not statistically significant. Future tobacco use exhibited a weak negative association with rsFC in the right SMH (r = −0.009, p = 0.352) and left CON (r = −0.022, p = 0.027), as well as a small but significant positive relationship with fun seeking (r = 0.051, p < 0.001). Similarly, future marijuana use showed a small positive association with fun seeking (r = 0.046, p < 0.001) but minimal relationships with rsFC or other variables.

Table 3 presents the covariances among baseline family income, NAcc rsFC with SMH, SMM, and CON, reward salience, and future substance use. Higher baseline family income was positively associated with baseline NAcc rsFC (B = 0.092, SE = 0.019, 95% CI: 0.054–0.130, p < 0.001) and negatively associated with baseline reward salience (B = −0.040, SE = 0.019, 95% CI: −0.078–−0.003, p = 0.036) and future substance use (B = −0.081, SE = 0.013, 95% CI: −0.107–−0.056, p < 0.001). Baseline NAcc rsFC was strongly and positively associated with baseline reward salience (B = 0.734, SE = 0.008, 95% CI: 0.717–0.750, p < 0.001) and future substance use up to age 13 (B = 0.124, SE = 0.013, 95% CI: 0.098–0.150, p < 0.001). Additionally, baseline reward salience was positively associated with future substance use up to age 13 (B = 0.176, SE = 0.010, 95% CI: 0.156–0.196, p < 0.001).

**Figure 1.**
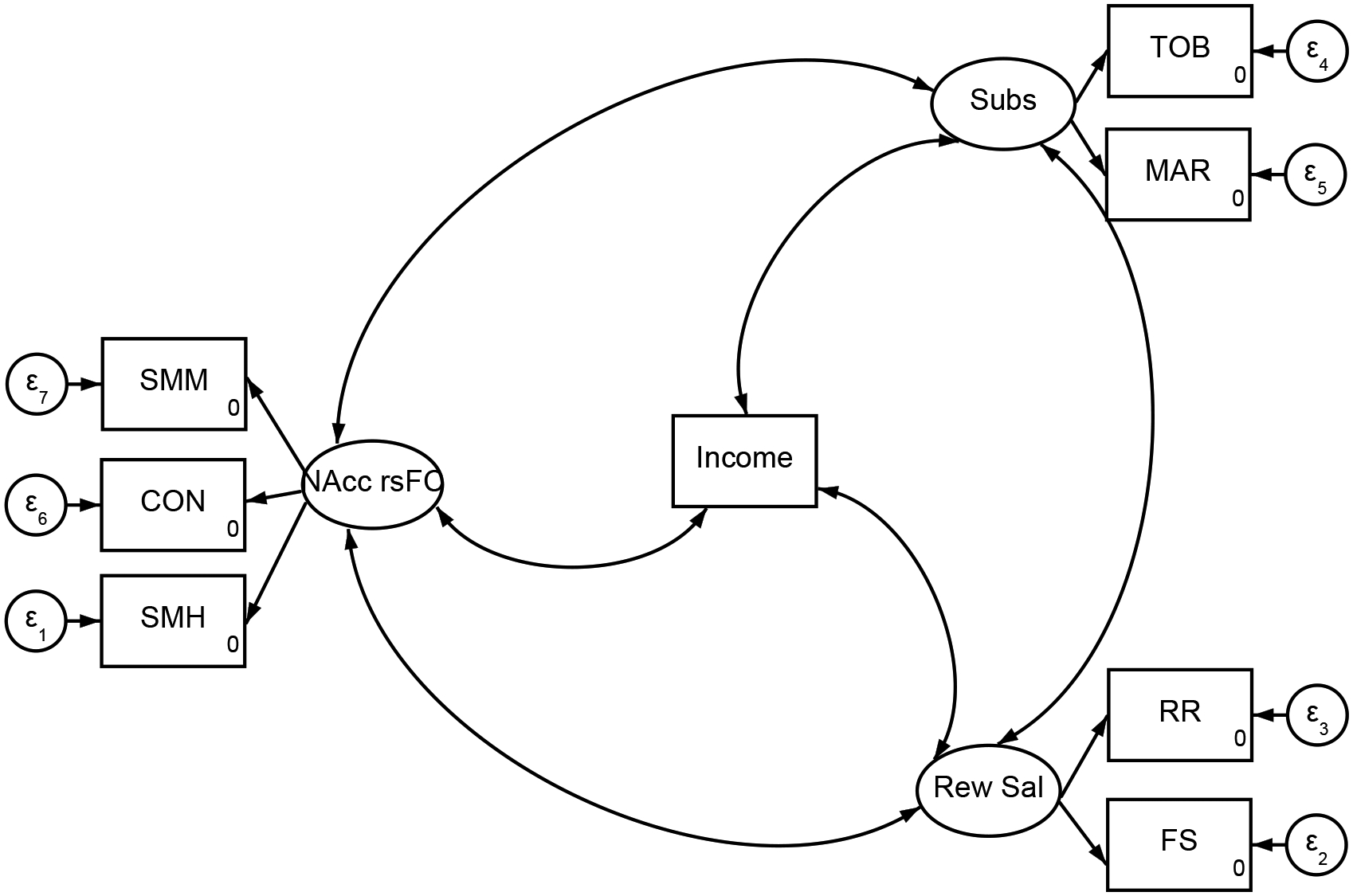
Structural Equation Model to Estimate Covariances between Family Income, NAcc rsFC, Reward Salience, and Substance Use *Note:* CON = Left Cingulo-Opercular Network; FS = Fun Seeking; Income = Family Income; MAR = Marijuana Use Initiation; NAcc = Nucleus Accumbens; Rew Sal = Reward Salience; RR = Reward Responsiveness; rsFC= Resting State Functional Connectivity; SMH = Right Sensorimotor Hand Network = SMH; SMM = Left Sensorimotor Mouth Network; Subs = Substance Use; TOB = Tobacco Use Initiation

Figure 1 illustrates the covariances among several key variables: baseline family income, the NAcc rsFC with the SMH, SMM, and CON networks, baseline reward salience, and future substance use. Higher family income at baseline was associated with stronger NAcc rsFC, yet it was linked to lower levels of reward salience at baseline and reduced substance use later on. Moreover, NAcc rsFC at baseline showed a robust positive relationship with both reward salience at baseline and substance use through age 13. Finally, higher reward salience at baseline was also positively correlated with subsequent substance use.

## 4. Discussion

This study explored the relationship between NAcc rsFC, reward salience, and substance use in adolescents, with a particular focus on the role of SES. Our findings provide important insights into the neural and behavioral mechanisms underlying substance use risk during adolescence, highlighting the influence of both environmental and neural factors.

### 4.1. Key Findings

The results showed that greater NAcc rsFC with cingulo-opercular, sensorimotor mouth, and sensorimotor hand networks was associated with heightened reward salience and increased future substance use. Low family income was linked to greater reward salience and elevated NAcc rsFC with cingulo-opercular, sensorimotor mouth, and sensorimotor hand networks, reinforcing the notion that socioeconomic disadvantage amplifies risk factors for substance use. These findings suggest that the NAcc, as part of the broader reward network, interacts with cognitive and motor regions to influence behavior, particularly in adolescents exposed to economic adversity.

It is already known that the NAcc plays a role in how SES influences the brain’s reward system, including reward salience and reward-seeking behavior related to addiction [49–51]. Our study suggests that the NAcc rsFC is associated with SES, reward salience, and substance use. These findings may indicate that differences in NAcc connectivity could be one of several factors linking social and economic conditions to variations in reward processing and substance use behavior.

### 4.2. Implications

These findings have several critical implications. First, the observed associations highlight the potential utility of NAcc rsFC as a neural marker for identifying adolescents at elevated risk for substance use, particularly those from low-SES backgrounds. Understanding how reward circuitry interacts with cognitive and motor networks can inform targeted prevention strategies. For example, interventions focused on reducing reward salience or enhancing cognitive control may mitigate substance use risk in vulnerable populations.

Second, these results underscore the broader impact of socioeconomic disparities on neural development and behavior. Addressing structural inequalities that contribute to heightened reward salience, such as chronic stress or limited access to resources, could reduce the incidence of substance use in low-income populations. Policies that enhance family stability, improve educational opportunities, and provide mental health support may have downstream effects on neural and behavioral health in adolescents.

### 4.3. Limitations

Several limitations of this study warrant consideration. First, the cross-sectional nature of the fMRI data limits causal interpretations. Longitudinal imaging data are needed to clarify the temporal dynamics of the relationships among SES, NAcc rsFC, reward salience, and substance use. Second, while family income was used as a proxy for SES, other indicators such as parental education, neighborhood characteristics, and financial stress were not included in this analysis and should be explored in future research. Finally, the sample was drawn from the ABCD study, which, while diverse, may not fully capture the socioeconomic and cultural heterogeneity present in broader populations.

### 4.4. Strenghts

The study possesses several significant strengths. First, the ABCD dataset provided an exceptionally large sample size, which is uncommon in brain imaging research given the high cost of MRI scans. Second, substance use was measured prospectively, and all participants were substance use–naïve at baseline, thereby reducing potential confounding effects. Third, our analytical approach utilized structural equation modeling (SEM), which enabled the simultaneous testing of multiple outcomes within a single model.

### 4.5. Future Research

Future studies should investigate the developmental trajectories of NAcc rsFC and its relationship with reward salience and substance use. Longitudinal research could elucidate causal pathways and identify critical periods for intervention. Examining potential moderators, such as resilience, peer influences, and access to mental health resources, could offer insights into protective factors that buffer against the effects of low SES and heightened reward salience. Additionally, advanced neuroimaging techniques, including graph theory, could be employed to further understand the organization of reward-related networks and their contributions to substance use behaviors.

## 5. Conclusion

This study advances our understanding of the neural mechanisms linking socioeconomic status, reward salience, and substance use in adolescents. The findings underscore the critical role of NAcc rsFC with diverse brain networks in mediating these relationships, providing a foundation for targeted interventions and policy development. Addressing both the neural and social determinants of health holds promise for reducing substance use disparities and promoting health equity among youth. Continued research is essential to refine these findings and translate them into effective prevention and intervention strategies.

## Figures and Tables

**Table 1.** Descriptive Data.

Variable	Mean	SE
Family Income	7.213	0.028
Reward Responsiveness	10.963	0.034
Fun Seeking	5.667	0.031
NAcc rsFC with the Right SMH	0.209	0.002
NAcc rsFC with the Left CON	0.098	0.002
NAcc rsFC with the Left SMM	0.195	0.003

*Note:* CON = Left Cingulo-Opercular Network; NAcc = Nucleus Accumbens; rsFC= Resting State Functional Connectivity; SMH = Right Sensorimotor Hand Network = SMH; SMM = Left Sensorimotor Mouth Network

**Table 2.** Bivariate Associations between Family Income, NAcc rsFC, Reward Salience, and Substance Use

	1	2	3	4	5	6	7	8
1 Family Income	1.000							
2 Reward Responsiveness	−0.102	1.000						
	<0.001							
3 Fun Seeking	−0.091	0.467	1.000					
	<0.001	<0.001						
4 NAcc rsFC with the Right SMH	0.110	−0.020	−0.049	1.000				
	<0.001	0.075	<0.001					
5 NAcc rsFC with the Left CON	0.053	−0.009	−0.009	0.251	1.000			
	<0.001	0.426	0.421	<0.001				
6 NAcc rsFC with the Left SMM	−0.007	0.028	0.015	0.114	0.175	1.000		
	0.503	0.012	0.182	<0.001	<0.001			
7 Future Tobacco Use	−0.046	0.013	0.051	−0.009	−0.022	−0.013	1.000	
	<0.001	0.212	<0.001	0.352	0.027	0.175		
8 Future Marijuana Use	−0.066	0.007	0.046	−0.001	−0.009	−0.021	0.446	1.000
	<0.001	0.528	<0.001	0.913	0.348	0.031	<0.001	

*Note:* CON = Left Cingulo-Opercular Network; NAcc = Nucleus Accumbens; rsFC= Resting State Functional Connectivity; SMH = Right Sensorimotor Hand Network = SMH; SMM = Left Sensorimotor Mouth Network

**Table 3.** Covariance between Family Income, NAcc rsFC, Reward Salience, and Substance Use

Variable 1	Variable 2	B	SE	95%	CI	p
Baseline Family Income	Baseline NAcc rsFC	0.092	0.019	0.054	0.130	< 0.001
Baseline Family Income	Baseline Reward Salience	−0.040	0.019	−0.078	−0.003	0.036
Baseline Family Income	Future Substance Use	−0.082	0.013	−0.107	−0.056	< 0.001
Baseline NAcc rsFC	Baseline Reward Salience	0.734	0.008	0.717	0.750	< 0.001
Baseline NAcc rsFC	Future Substance Use	0.124	0.013	0.098	0.150	< 0.001
Baseline Reward Salience	Future Substance Use	0.176	0.010	0.156	0.196	< 0.001

*Note:* NAcc = Nucleus Accumbens; rsFC= Resting State Functional Connectivity
